# The Aesthetic Appreciation of Multi-Stable Images

**DOI:** 10.3390/jimaging11040111

**Published:** 2025-04-04

**Authors:** Levin Saracbasi, Heiko Hecht

**Affiliations:** Department of Psychology, Johannes Gutenberg-Universität Mainz, 55122 Mainz, Germany; levin.1998@yahoo.de

**Keywords:** multi-stable perception, aesthetics, processing fluency, visual ambiguity

## Abstract

Does the quality that renders multi-stable images fascinating, the sudden perceptual reorganization, the switching from one interpretation into another, also make these images appear beautiful? Or is the aesthetic quality of multi-stable figures unrelated to the ease with which they switch? Across two experiments, we presented multi-stable images and manipulated their perceptual stability. We also presented their unambiguous components in isolation. In the first experiment, this manipulation targeted the inherent stimulus stability through properties like figural size and composition. The second experiment added an instruction for observers to actively control the stability, by attempting to either enhance or prevent perceptual switches as best they could. We found that higher stability was associated with higher liking, positive valence, and lower arousal. This increase in appreciation was mainly driven by inherent stimulus properties. The stability instruction only increased the liking of figures that had been comparatively stable to begin with. We conclude that the fascinating feature of multi-stable images does not contribute to their aesthetic liking. In fact, perceptual switching is detrimental to it. Processing fluency can explain this counterintuitive finding. We also discuss the role of ambiguity in the aesthetic quality of multi-stable images.

## 1. The Fascination Behind Multi-Stable Stimuli

**Figure 1:**
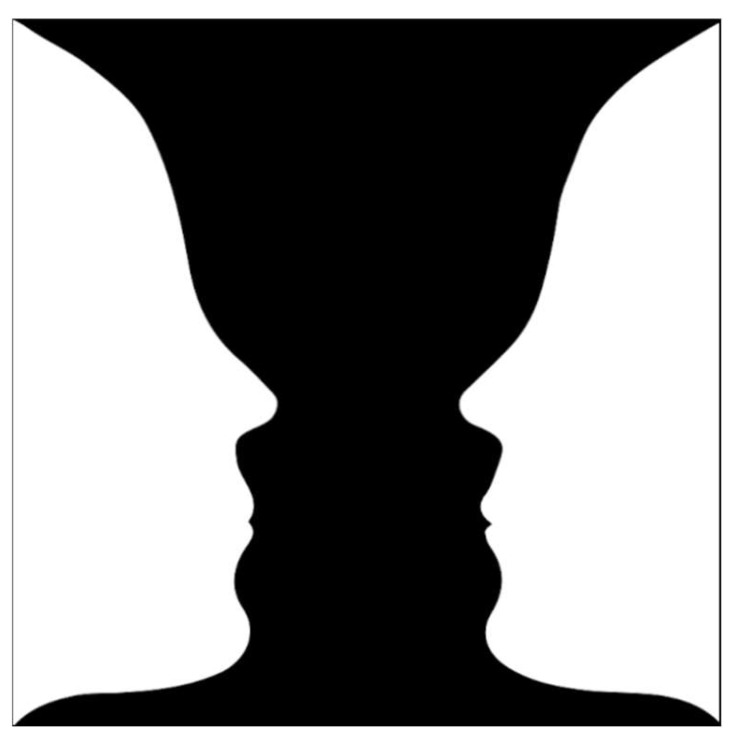
Rubin’s vase can be seen as two faces or one vase.

Multi-stable figures like the Necker cube, the duck/rabbit, or Rubin’s vase (see Figure 1) evoke two different unique interpretations which arise in sequence, but never simultaneously [1,2]. The fact that the percept can spontaneously switch from one internally consistent interpretation to the other gives these figures an unresolvable ambiguity [3]. These multi-stable figures have been an object of scientific inquiry for over a century [4], and they have inspired artists such as M. C. Escher, Octavio Ocampo, and Salvador Dali, among many others. Since ambiguity has frequently been linked to aesthetic appreciation (see for example [5,6,7]), we asked whether the perceptual instability of multi-stable figures impacts their aesthetic liking and, if so, through what mechanism.

To answer this question, we isolated and manipulated the stability of different stimuli and investigated the resulting aesthetic appreciation. Across two experiments, participants saw different virtual multi-stable figure–ground illustrations and rated them on their liking, arousal, and valence. The first experiment was focused on altering the stability through inherent stimulus properties, while the second experiment involved an additional instruction to either facilitate or prevent perceptual switching. We have chosen bi-stable figure–ground stimuli akin to Rubin’s vase, since manipulating the dividing border between both figures serves as an effective tool to vary stimulus stability. We assume that processing fluency will drive many of the effects [8], with the hypothesis that higher instability emphasizes ambiguity within the stimuli, thereby lowering subjective processing ease. In the following, we discuss figure–ground assignment, multi-stability and its experimental manipulation, and the different implications that ambiguity may have on aesthetic appreciation. We then report two experiments to show how stability affects aesthetic judgements.

### 1.1. Figure–Ground Organization

Reversable figure–ground images represent a sub-category of multi-stable perception, in which an unchanging stimulus can evoke two different interpretations. While either of these interpretations assembles the given information in a coherent and comprehensive way, they are both mutually exclusive, which is what constitutes their ambiguous quality [2]. As illustrated by Rubin’s vase, the quality of ambiguity within these images arises because adjacent areas do not have a clearly defined figure–ground relationship [9]. The process of figural assignment depends on the dividing border between both areas [10]. When one area gets assigned a figural status, it is seen as owning the border, thereby incorporating it into its shape as an outer edge. This also leads to depth perception, since the figure seems to occlude the other area, which is perceived as a shapeless background [11]. Importantly, this multi-stable percept can spontaneously reorganize itself, such that a sudden switch in figural assignment causes a switch in interpretation of the image [12,13].

As Wagemans et al. [14] outlined, the initial investigation into figure–ground segmentation started with Gestalt psychologists, who identified many properties which increase the probability of figural assignment. For example, an area is more likely to be seen as a figure when it is convex, symmetrical, or enclosed by another area. More recently, other properties have been identified: figure assignment is more likely for an area if it is wider at the bottom [15], is lower within the frame [16], has extremal edges [17], is familiar to the observer [11], includes certain movement-based cues [18], or when exogenous attention is focused upon it [19]. Attention is not necessary for figure–ground processing, the segmentation can even occur without overt realization by the observer [20]. For the presence of multiple cues, Devinck and Spillmann [21], for example, infer that a comprehensive model would likely incorporate the sum of cooperating and opposing figural cues in a weighted fashion.

Conceptually, figure–ground organization is assumed to work according to a winner-takes-all inhibitory competition model. Salvagio et al. [22] argue that once a figure is decided upon, an increase in activity of visual units stabilizes the Gestalt of the figure, while neuronal suppression on the opposite side of the border makes the ground seem shapeless. They show that after a figural status is assigned, an observer’s identification of a stimulus on the ground side close to the mutual border becomes poorer as the figural competition increases, supporting the idea of temporary ground suppression. Modern models generally assume that during the initial processing, object recognition can take place even before figure–ground relationships are fully processed [23,24,25].

### 1.2. Assessing Stability of Multi-Stable Figures

Within multi-stable figure–ground constellations, Pelton et al. [26] define stability as the duration for which one perceptual organization is upheld, in other words the interval between switches, and attraction as the probability that a given percept is perceived at any given moment. As these concepts overlap, we will refer to stability to describe the essence of both of these aspects. Strüber and Stadler [27] outline how a common explanation for the alternating perception in multi-stable stimuli, originally proposed by the Gestalt psychologist Wolfgang Köhler in 1940, is the concept of neuronal satiation. As we continue to look at an ambiguous stimulus, the neuronal system responsible for upholding the dominant interpretation fatigues to the point of collapse. Since the figure assignment is decided by the highest corresponding neuronal firing rate [28], when one neuronal representation gets fatigued enough, the other interpretation starts to dominate until the next switch occurs. This theory of cyclical fatigue and incomplete recovery also explains the fact that the re-organization cycles get shorter with each switch [27,29]. This classic bottom-up explanation has been supplemented with more active, cognitively driven, top-down approaches. Leopold and Logothetis [30] raise different empirical findings that support such active control: perceptual switches in part underlie voluntary control (especially when figures contain semantic meaning, see [27]), they are influenced by the observer’s mood and attention, the process can be practiced, their temporal sequence resembles actively initiated behaviors, and brain areas relevant for many cognitive behaviors are active during the processing of multi-stability. Strüber and Stadler [27] add that the alternations depend on whether or not the observer is aware of the multiple perceptions of the stimulus (see also [31]). In an experiment aimed at differentiating the two types of automatic and voluntary alternations, Long and Moran [32] showed that a short prime can make a given figure more dominant (making the representation more readily available), whereas longer priming makes the opposite figure more dominant (by pre-fatiguing one of the representations). They take this to suggest a hybrid model of both bottom-up and top-down integration for reversals in multi-stable stimuli.

### 1.3. Ambiguity of Multi-Stability

Kaplan and Kris [33] define something as ambiguous when it cannot be confined to a single distinct meaning, but rather evokes a cluster of multiple possible interpretations. Note that this does not include paradoxical or indeterminant figures, because they either evoke only a single seemingly correct interpretation despite a mismatch with the given visual information (like the Fraser spiral), or they elude a single coherent interpretation because of their paradoxical nature (like the devil’s pitchfork) [34]. In contrast, multi-stable figures are ambiguous because even though they evoke multiple interpretations, all of them coherently incorporate the given visual information, albeit in different ways [34]. Kaplan and Kris [33] lay out four different types of ambiguity: disjunctive (mutually exclusive meanings), additive (exclusive, but overlapping meanings), conjunctive (both meanings simultaneously affect the interpretation), and integrative (both meanings combine into one unified interpretation). Elaborating this taxonomy, Tormey and Tormey [34] have described disjunctive ambiguity within the visual realm as containing three sub-categories: depth ambiguity (like the Necker cube), objective ambiguity (like the duck/rabbit), and figure–ground ambiguity (like Rubin’s vase).

Whereas all of these categories involve perceptual switches, when trying to experimentally investigate what influence this quality has on aesthetic appreciation, the challenge is to isolate and manipulate the property of perceptual stability, while at the same time preserving the ambiguity of a stimulus. Additionally, this manipulation has to be continuous and largely independent of other variables. To this end, we have chosen multi-stable figure–ground stimuli, because the dividing border between the figures can be freely modulated. Considering the prior figural assignment cues, by varying the shapes and compositions of the figures, we can produce multiple constellations that result in multiple levels of stability.

### 1.4. Aesthetic Appreciation of Perceptual Switching

To our knowledge, stability or perceptual switching have not previously been experimentally related to aesthetic appreciation, but more general concepts have. Complexity (e.g., [35,36]), novelty (e.g., [37,38]), and ambiguity (e.g., [6,7]) have all been identified as influential factors that shape aesthetic appreciation. More specifically, ambiguity is often described as having a positive impact on liking when it is accompanied by a subjective feeling of insight, or when it can act as a vehicle to add new layers of meaning to an artwork [39,40,41,42]. A particularly illustrative case for gaining appreciation through insight is the aesthetic Aha effect. It describes a sudden aesthetic revelation where information is (re-)assembled into a new coherent Gestalt, accompanied by feelings of suddenness, ease, positive affect, and conviction of truth [43]. Muth and Carbon [44], for example, showed that for sudden discoveries of faces within ambiguous illustrations, the insight produces a direct, albeit temporally limited, effect on liking. It is assumed to be inherently pleasurable and relaxing to achieve mental closure and cognitive resolution after having experienced ambiguity [45], a process that has been described by Kenning et al. as informational simplification leading to “cortical relief” (as cited by [46]; see also [47]). Muth and Hesslinger [42] noted that appreciation does not depend on fully resolving ambiguity, but rather on the strength of the insight that is gained during elaboration.

For the analysis of multi-stable figures, it is also helpful to differentiate aesthetic pleasure from aesthetic interest. For example, Turner and Silvia [48] found that disturbing pictures can be interesting but unpleasant, with the opposite effect occurring for calming pictures. They showed pleasure and interest to be largely unrelated constructs, with complexity and novelty positively predicting interest, but negatively predicting pleasure. Graf and Landwehr [49] posit that aesthetic pleasure involves an immediate, gut-level reaction that is mostly stimulus driven, whereas aesthetic interest arises through more active engagement and the desire for cognitive enrichment. This is mirrored by Silvia [50], who describes interest as a knowledge-based emotion embedded into appraisal theory. Interest is posited to include two central subjective assessments of the observer: first, appraisal that a stimulus holds new information (novelty check), and second, feeling capable of gaining insight into the given ambiguity (coping potential). The research on aesthetic liking, in contrast, is more often involved with stimulus features or with how easily the stimulus is processed, in turn making it appear beautiful as opposed to interesting [8,51]. We take interest in multi-stability as given and thus focus on how the property itself interacts with the dimension of aesthetic liking.

### 1.5. Processing Fluency

One of the pre-dominant attempts to predict aesthetic appreciation is the hypothesis that pictures which are easy to process and to grasp perceptually and cognitively are liked better than those that are not. This theory of processing fluency contains four main assumptions: some stimuli can be processed more easily than others; high perceptual fluency is in itself positively hedonically marked (but see also [52]); this feeling of (dis)fluency feeds into a subjective experience which is assessed for an evaluation of the stimulus; and the attribution and expectation of feelings of fluency influence the impact on the observer [8]. The subjective feeling of fluency has been shown to be a better predictor of aesthetic appreciation than is fluency as defined by stimulus manipulation itself, and observers should be able to explicitly report their respective feelings of fluency [53]. The theory is as parsimonious as it is potent, with its reach spanning beyond aesthetic explanations. Things that are easier to process not only seem more beautiful [8], they are also judged as more truthful [54], more familiar (and thus less risky) [55], and are more likely to be purchased [56].

Adding ambiguity to a stimulus makes it more difficult to process. On a fundamental level, the aim of the visual system is to remove ambiguity from a given input [57], such that the observer can reach an interpretation of the percept which is most likely to be a correct representation of the surrounding world [58]. In the case of an aesthetic Aha experience, such as when suddenly finding a hidden Gestalt within visual noise [44], processing fluency is regained through the resolution of the prior ambiguity. The ambiguity within multi-stability poses a more sustained perceptual challenge, because a multi-stable percept signals to the observer that there are two possible interpretations of the given information, which are equally likely to represent the surroundings accurately. Therefore, one interpretation cannot easily be discarded in favor of another in an effort to conclusively solve this perceptual challenge [3,58]. Since the visual system continues to probe different solutions to a multi-stable stimulus, an observer only ever experiences apparent perceptual stability [2] that is temporally limited, because there is always another coherent way to assemble the information.

Thus, we posit that the way in which multi-stability can lower processing fluency is two-fold. Regarding the cortical levels where the image is processed and represented [4], every switch should involve a disruption of the perceptual process, including a need for readjustment to the new interpretation, thereby lowering fluency. Additionally, every perceptual switch also emphasizes the fact that, since the information can always be assembled in another equally valid way, any single interpretation fails to fully grasp the informational content of the stimulus and thus, at least in some part, remains inadequate. Therefore, because every switch should make this ambiguity more salient to the observer, more frequent switches should lower the subjective feelings of fluency even further.

Our resulting hypothesis may seem somewhat counterintuitive. We predict that the less stable a multi-stable stimulus is, the lower its aesthetic appreciation. Thus, according to fluency theory, what makes these stimuli fascinating in the first place, their perceptual instability, should be detrimental to their aesthetic quality. We hypothesize that the liking of a multi-stable stimulus is increased by reducing or removing its ambiguous quality altogether, which makes it easier to process, and thus, more beautiful.

There are two different sources that determine the resulting stability of a stimulus, both of which can be manipulated independently: perceptual switches can stem either from inherent stimulus properties or from an observer who imposes active perceptual control upon the stimulus [27]. In order to test if and how these two sources impact liking via processing fluency, we conducted two different experiments in which we manipulated the stability of multi-stable stimuli. The first experiment focused on stimulus properties. We presented different multi-stable stimuli with inherently higher or lower stability, as well as their composing figures in isolation, and let observers rate them on liking, arousal, and valence, as well as on subjective stability. In the second experiment, we added an additional instruction for the observers to actively control the stimulus stability and asked them to attempt either to enhance or to prevent perceptual switching as much as they could. Both experiments were conducted with stimulus material that was originally constructed by Kuo et al. [59] (we thank the authors for granting us access to their multi-stable stimuli and their components), who utilized their own original algorithm to produce a large set of multi-stable images. All multi-stable figures, as well as the corresponding isolated components that were used in this study, are accessible in our online Supplementary Material at https://osf.io/msr67/ (accessed on 3 April 2025).

## 2. Experiment 1

The first experiment focused on manipulating stability through inherent stimulus properties, and how this affects aesthetic appreciation. The stability manipulation was achieved by choosing a sample of different bi-stable images, which included varying figural sizes, shapes, and compositions, while keeping other stylistic elements, as well as complexity and color, constant. The resulting dividing borders contained different ratios of figural assignment cues for the adjacent areas, which led to a natural variation in stability across the stimuli. In a within-subjects design, the participants saw every bi-stable illustration consecutively, as well as stimuli that merely contained the figural parts in isolation. They rated them once on liking, arousal, and valence, and once on subjective stability. The subjective stability ratings were used to split the multi-stable stimuli into high- and low-stability groups for subsequent analysis. We chose to use liking, as opposed to interest, as a measure of subjective beauty. Since processing fluency was shown to go along with positive affect [60], more stable stimuli should produce greater liking and more positive valence, as compared to less stable stimuli. Moreover, stable stimuli should produce lower arousal as they are easier to process and require fewer cognitive resources and less effort.

Thus, according to the notion of processing fluency, we expected the most stable stimuli to elicit the highest aesthetic pleasure. We hypothesized that out of the three stability groups, isolated figures would produce the highest aesthetic appreciation, followed by multi-stable stimuli with high stability. Highly unstable multi-stable stimuli should produce the lowest liking scores. Aesthetic appreciation should be captured by high liking and low arousal. We also asked for valence ratings to assess the observer’s emotion induced by the stimuli. They are not necessarily the same as liking scores, as it is conceivable that observers might appreciate an artwork at a purely intellectual level without feeling a positive emotion.

### 2.1. Method

The experiment was conducted as an online study using the platform *SoSciSurvey.de* (Version 3.5.01) [61].

#### 2.1.1. Participants

A total of 52 students (16 men, 35 women, 1 non-binary) provided informed consent and completed the entire experiment. They were mostly German psychology students at the Johannes-Gutenberg University Mainz and could receive partial course credit for their participation. Their ages ranged from 19 to 60 years (*M* = 29.19, *SD* = 1.57). The only participation criteria were self-reported German skills and visual acuity.

#### 2.1.2. Stimuli and Material

The stimuli used for the experiments were originally created by Kuo et al. [59]. They devised an algorithm that matches the contours between two or more figures, smooths out the resulting border between the figures while preserving the original figural contours, and, through a final crop and binarization, automatically creates a black-and-white multi-stable stimulus. The functionality of the perceptual switching for the resulting images had also been verified by Kuo et al. [59]. For a more detailed description of the algorithm parameters regarding stimulus generation, we refer to their original paper. We selected 22 bi-stable stimuli and 33 corresponding isolated figures out of these stimuli for our sample, such that half of them were of high or low stability, respectively. Note that some isolated figures appeared multiple times in different multi-stable stimuli, such that the number of figures was not double the number of multi-stable images. All the stimuli contained salient shapes of silhouettes, such as profiles of human faces or animals. This should help observers control perceptual switches more easily [27], which will become relevant in the second experiment.

Next, the stimuli were adjusted using the editing software *GIMP* (Version 2.10.36) [62], such that the figures and ground consisted of a standardized shade of black or white. The values of the shades were set, respectively, to a minimal or maximal lightness. We also balanced, as well as possible, absolute and relative total color area across all stimuli. The stimuli were controlled to have broadly equivalent size, because size differences influence the frequency of perceptual switching [4,27]. The figures were adjusted in terms of shape and orientation to match their corresponding multi-stable stimuli, and, since the web browser had a white background, a slim black frame was added to all stimuli. The resulting material exhibited stark similarity and simplicity regarding its stylistic composition. The identical stimuli were used for both experiments without any subsequent alterations. All stimuli are illustrated in Appendix A and are accessible in their original sizes and resolutions in our Supplementary Material under https://osf.io/msr67/. Since the participants were free to choose the device on which they completed the online questionnaire, parameters like screen brightness, viewing angle, or screen distance could not be controlled. However, we encouraged participants to use an adequately large screen and to adjust their browser zoom such that the stimuli were entirely displayed.

#### 2.1.3. Design

Every participant rated each stimulus, which resulted in a within-subjects design with repeated measurements. The independent variables were inherent stimulus stability (three factor levels: multi-stable with high stability, multi-stable with low stability, and isolated figures) and specific stimulus (11 unique stimuli for each of the two stimulus groups). Four dependent variables were collected (liking, arousal, valence, and subjective stability ratings).

#### 2.1.4. Procedure

The stimuli were presented in different individual random orders and required about 20 min to be judged. Following an instruction slide and a few demographic questions, a version of Rubin’s vase was presented as a training stimulus, which served to briefly explain the concept of figure–ground multi-stability and gave an opportunity to get accustomed to perceptual switching. The participants first rated a block containing 22 multi-stable and 33 isolated stimuli, which were presented one at a time. They were explicitly told that in this block, a given segment contained either only multi-stable stimuli or only isolated figures. The participants could view the stimulus as long as they wished.

Underneath each stimulus, three sliders were presented, one each to indicate liking, arousal, and valence. Liking measurements were operationalized as beauty judgements (see [63]) with the question “How beautiful do you judge the image above to be?”, assessed with a Likert scale slider coding for 1 (not beautiful at all) to 101 (very beautiful), with the selected rating number displaying above the slider. The original German versions of the questions, as embedded into the original slides of the questionnaire, are illustrated in Appendix B. Arousal and valence ratings were operationalized through self-assessment manikins (SAMs) [64], with the five different figural-style Likert scale options, and their intermediate points, coded from 1 (very low) to 9 (very high). The respective questions were “How relaxed or aroused do you feel regarding the image above?” and “How good or bad do you feel regarding the image above?”. The SAM scales only displayed the corresponding manikins, with no additional text or numbers added. It was specified that the ratings relate to the emotion the observer feels in that moment, not the emotion they perceive in the stimulus. Additionally, the participants were encouraged to answer intuitively and from a personal perspective, disregarding how other people might feel.

In the second block, the participants again saw the multi-stable and isolated figures one at a time and judged them on their subjectively perceived stability. The participants were told that in this segment, a mix of multi-stable stimuli and isolated figures would be presented. Stimulus stability ratings were conducted through a subjective question beneath each image (“Relative to the other images—how often do the figures in the image above switch from foreground to background and vice versa?”), with a Likert scale slider coding for 1 (never) to 101 (very often). Note that since this question represents the frequency of perceptual switches, a higher score represents higher levels of instability.

The participants always saw the block for aesthetic appreciation first, and the subjective stability block second. We chose this order so that the ratings of liking were free of potential mere-exposure confounds, while simultaneously letting the participants get accustomed to the stimuli switches before rating stability. Both blocks were concluded with a rating of perceived difficulty and exertion of the task, which applied to the entire block. Finally, there was space to leave a comment.

### 2.2. Results

#### 2.2.1. Stability

First, we sorted the multi-stable stimuli into high- and low-stability groups according to the subjective stability ratings by the participants. This was conducted through a median split for the mean ratings for each stimulus. This group split was additionally validated by the researchers through a pre-assessed group categorization of the stimuli, as determined by their anticipated stability. This categorization was largely in line with the median-split results, which were adopted for the data analysis. The stability ratings of the stimuli in each group, including the corresponding liking ratings for both experiments, are detailed in Appendix A.

When comparing multi-stable to isolated figures, the latter, not surprisingly, were judged to be the most stable. Note that for the stability measures, the participants rated the frequency of perceptual switches. Thus, higher ratings mean that a stimulus has higher instability. Descriptively, the low-stability images (*M_Low_* = 71.06, *SD_Low_* = 21.87) were rated as noticeably less stable than the high-stability images (*M_High_* = 62.70, *SD_High_* = 20.75), with the group of isolated figures expectedly showing the least instability (*M_Fig_* = 9.62, *SD_Fig_* = 12.96). Stability showed a large significant main effect (*F*(1.22, 62.20) = 259.66, *p* < 0.001, *η_p_²* = 0.836) (note that degrees of freedom were Greenhouse–Geisser corrected in all ANOVA results where appropriate). In the pairwise comparisons, all the groups were different from one another, with the higher multi-stable group producing significantly fewer switches than the lower group (*p* < 0.001, *M_Diff_* = −8.36, 95% CI [−11.70, −5.03]). Thus, the group split can be used to investigate whether stimulus stability causes differences in liking, arousal, and valence. For the following results, note that liking was measured on a scale from 1 to 101, while arousal and valence involved a scale from 1 to 9.

All the calculations were conducted using *IBM SPSS Statistics* (Version 29.0) [65], for which all of the 52 participants were included. We conducted an rmANOVA with the independent variable of inherent stimulus stability (three factor levels: multi-stable with high stability, multi-stable with low stability, and isolated figures) and the independent variable of specific stimulus (11 individual stimuli per stability group), for all dependent variables (four measures: liking, arousal, valence, and subjective stability). To control for confounding effects, the rmANOVA was additionally conducted with the between-factor of gender. Additionally, the covariance of liking and valence was assessed through a linear regression model across all stimuli. As a control measure, different multiple linear regressions were calculated, where the measures of liking, arousal, valence, and subjective stability were each predicted through perceived exertion and subjective adequacy of task completion in the given block. In order to match the amount of 11 multi-stable stimuli in the other two groups, the 33 isolated figural components were condensed into 11 figural averages using the data points of three figures each.

#### 2.2.2. Liking

**Figure 2:**
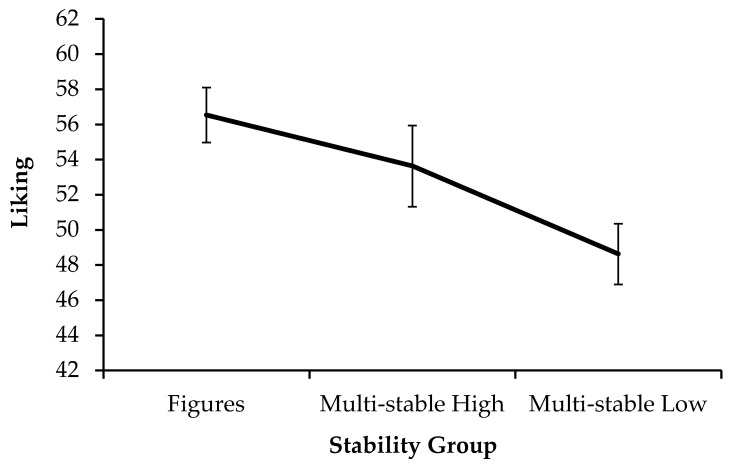
Liking ratings for the three stimulus groups of isolated figures, multi-stable stimuli with high stability, and multi-stable stimuli with low stability (Exp. 1). Error bars show standard errors of the mean.

As is visible in Figure 2, liking increased with stability, from multi-stable images with low stability (*M_Low_* = 48.62, *SD_Low_* = 12.46), to those with high stability (*M_High_* = 53.63, *SD_High_* = 16.67), to isolated figures (*M_Fig_* = 56.54, *SD_Fig_* = 11.26). An rmANOVA on the liking scores showed a main effect of stability (*F*(1.70, 86.67) = 10.55, *p* < 0.001, *η_p_²* = 0.171). Pairwise comparisons revealed that this effect could mainly be ascribed to the low-stability group. Multi-stable stimuli with high stability were liked significantly more than the unstable ones (*p* = 0.004, *M_Diff_* = 5.00, 95% CI [1.37, 8.64]). The isolated figures were not different from the multi-stable images with high stability (*p* = 0.496, *M_Diff_* = 2.91, 95% CI [−2.20, 8.03]).

#### 2.2.3. Arousal

**Figure 3:**
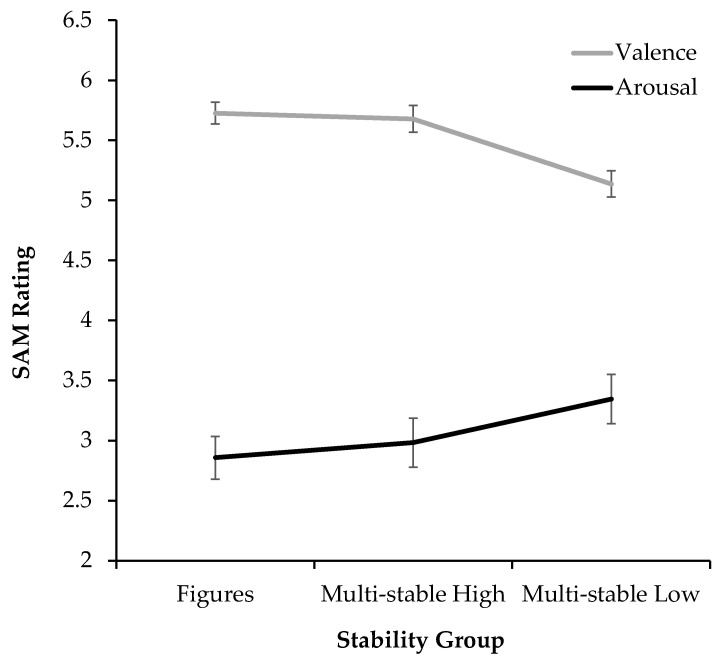
Arousal and valence ratings (made using the respective SAM scales) for the three stimulus groups of isolated figures, multi-stable stimuli with high stability, and multi-stable stimuli with low stability (Exp. 1). Error bars show standard errors of the mean.

In contrast to liking, arousal decreased with rising stability (see Figure 3), from multi-stable images with low stability (*M_Low_* = 3.35, *SD_Low_* = 1.47), to those with high stability (*M_High_* = 2.98, *SD_High_* = 1.47), to isolated figures (*M_Fig_* = 2.85, *SD_Fig_* = 1.29). For the measure of arousal, a large effect size was reached with a high level of significance (*F*(1.60, 81.75) = 11.16, *p* < 0.001, *η_p_²* = 0.180). Again, pairwise comparisons reveal that this effect is mainly due to the multi-stable low-stability group producing higher arousal than the other groups. The difference between the more stable multi-stable stimuli and the isolated figures failed to reach significance (*p* = 0.691, *M_Diff_* = 0.13, 95% CI [−0.13, 0.38]).

#### 2.2.4. Valence

As illustrated in Figure 3, valence was lowest for the multi-stable images with low stability (*M_Low_* = 5.14, *SD_Low_* = 0.79) and equally high for those with high stability (*M_High_* = 5.68, *SD_High_* = 0.81) and isolated figures (*M_Fig_* = 5.73, *SD_Fig_* = 0.65). The results also show a large effect size with high significance (*F*(2, 102) = 16.25, *p* < 0.001, *η_p_²* = 0.242). Pairwise comparisons show that the effect is carried by the multi-stable images with low stability. While the multi-stable stimuli produce a more positive valence than those with lower stability (*p*< 0.001, *M_Diff_* = 0.54, 95% CI [0.26, 0.83]), the multi-stable stimuli show no difference when compared to isolated figures (*p* = 1.000, *M_Diff_* = −0.05, 95% CI [−0.30, 0.21]).

#### 2.2.5. Covariation of Valence and Liking

As explicit liking and the emotional dimension of valence are likely to overlap, we computed a regression between the two measures over every rated stimulus (combining both multi-stable and isolated figures). The linear regression model proved to be highly significant (*F*(1, 50) = 26.70, *p* < 0.001). Valence explained about 33% of the variance in liking (*R²_adj_* = 0.335) and showed an increase in liking by 11.23 (95% CI [6.87, 15.60]) for each rating increase in valence by 1.

#### 2.2.6. Control Measures

When adding gender as a covariate in the rmANOVA, as described above, the variable did not reach significance. In the rmANOVA, the stimulus factor reached significance for liking (*F*(6.99, 356.80) = 9.20, *p* < 0.001, *η_p_²* = 0.153). To illustrate the variance in liking, especially between the individual multi-stable stimuli, we listed each image, including their corresponding liking and stability ratings, in the Appendix A.

Additionally, when calculating different linear regressions by using the measures of perceived adequacy of task completion and subjective exertion to predict ratings of liking, arousal, valence, and subjective stability, we found a small but significant relationship between valence and stability with perceived exertion. When only including perceived exertion as a predictor, a model for valence (*F*(1, 50) = 9.03, *p* = 0.004) explained about 14% (*R*²*_adj_* = 0.136) of the variance and showed a decrease in valence by −0.35 (95% CI [−0.59, −0.12]) for each rating increase in exertion by 1. For stability, a significant model (*F*(1, 50) = 9.09, *p* = 0.004) explained about 14% (*R*²*_adj_* = 0.137) of the variance, and showed a decrease in ratings of stimulus instability by −9.44 (95% CI [−15.73, −3.15]) for each increase in rated exertion by 1. Note that exertion was coded on a scale from 1 (low) to 4 (very high).

### 2.3. Discussion

We hypothesized that stable images produce higher aesthetic appreciation compared to unstable ones. This was indeed the case. In particular, liking (Figure 2) showed an increasing trend from multi-stable figures with low stability, to those with higher stability, to unambiguous figures in isolation. These results are in line with a processing fluency account, which would predict that frequent perceptual switches disrupt fluent sensory and cognitive processing, as well as make perceptual ambiguity more salient. Higher stability would thus lead to easier processing, which entails an overall bias towards positive stimulus assessment (in this case, higher liking and positive valence). The high fluency of more stable images would also go together with greater relaxation during processing, which is consistent with the lower arousal ratings.

Descriptively, isolated figures produced the highest liking ratings. Whereas the increase in appreciation from the lower-stability multi-stable group to the higher one was significant for every measure, the contrasts between highly stable multi-stable images and their isolated figures did not reach significance. However, especially when comparing both multi-stable groups with different levels of stability, enhancing the frequency of perceptual switches lowered appreciation. This suggests that the defining quality of multi-stable images is in fact detrimental to their aesthetic quality.

Arousal and valence (Figure 3) function as good indicators of why perceptual fluency might be a likely explanation for this result. The group of isolated figures depicts the same figures that make up the multi-stable stimuli, with the only difference lying in their compositional arrangement. Thus, if these affective ratings were to reflect an independent assessment of the recognized figure, the isolated stimuli would show a similar judgement irrespective of their compositional arrangement in a multi-stable image. It is therefore noteworthy that these figures, when compared to their isolated ratings, only produced more negative scores when assembled in an unstable composition, as opposed to a stable one. This discrepancy can be explained by their higher fluency, since easier processing also involves a mild, positive affective judgement, which is ascribed to the stimulus [60].

The finding that the contrasts between multi-stable images with high stability and their isolated figures did not reach significance could have two sources. On one hand, it is possible that additional mechanisms besides fluency, like insight and complexity, influence the aesthetic appreciation of multi-stability. On the other hand, since there was a trend indicating that the isolated figures were the most pleasing, the contrasts might have been masked by high variance in the aesthetic data. The second experiment was designed to further illuminate the role of stability.

## 3. Experiment 2

The second experiment added an external stability manipulation through observer instructions. We asked participants to make an effort to bring perceptual switches under voluntary control, and to either enhance the frequency of switches or prevent them entirely if possible. Our hypothesis was that the stability of any source, stemming from either inherent stimulus properties or cognitive manipulations, would increase the appreciation of a given stimulus. By assessing both sources of stability simultaneously, we would also find out whether they interact with each other. For example, for a stimulus that is already inherently unstable, it might be easier to enhance switching even further than it would be to hold it completely stable. Therefore, if a stable instruction gets combined with an inherently stable stimulus and vice versa, the resulting effect on stability might get reinforced, thereby also potentially amplifying any aesthetic outcomes. According to perceptual fluency theory, any resulting instability should be detrimental to aesthetic appreciation, regardless of whether it is due to a stimulus property or an instruction. As in the first experiment, higher appreciation should express itself through higher liking and more positive valence. If fluency drives arousal, the latter should be lower for stable stimuli.

### 3.1. Method

#### 3.1.1. Participants

A total of 84 (70 women, 14 men) participants performed Exp. 2, 20 of whom reported that they had also participated in the first experiment. Seventy-eight participants were enrolled as college students. The ages ranged from 19 to 55 years (*M* = 24.64, *SD* = 6.45).

#### 3.1.2. Design and Stimuli

The process of data collection was equivalent to that of the first experiment, with the exception of the instruction to either suppress or encourage multi-stability. A three-factorial design was fully crossed within subjects: inherent stimulus stability had two levels (high and low). For each level, the same 11 stimuli as in Exp. 1 were presented. Since inherent stability values for the stimuli could be adopted from the first experiment, no additional measurements to this end were collected in the questionnaire. We presented all of these stimuli once with the instruction to prevent reversals and once with the instruction to facilitate reversals as much as possible (high vs. low instructed stability). This was conducted in counterbalanced blocks. Within each block, the stimuli were ordered randomly. Each block consisted of the same 22 multi-stable stimuli, including both high and low inherent stability. As before, three dependent variables were collected (liking, arousal, and valence).

All the multi-stable stimuli of Exp. 1 were used. Since the instruction regarding controlling perceptual switches is not feasible for isolated figures, and to not overburden the participants, the isolated stimuli were omitted in Exp. 2. However, to help the participants maximally achieve the instructed stability, we briefly primed the interpretation of the multi-stable stimulus that should be held dominant. For this priming procedure, the isolated figures were used. Note that both the prime and target stimuli had been controlled regarding their absolute and relative ratios for black and white figures. They had also been adjusted in size and orientation to more closely match the following targets. To ensure the technical functionality of the priming procedure, the participants could only complete the questionnaire on a desktop.

#### 3.1.3. Procedure

The procedure was identical to that of Exp. 1, with the exception of the added instructions to manipulate stability. Strüber and Stadler [27] showed that participants can aptly control switches, especially when given semantic content within the images upon which to focus. Before each block, an introductory slide explained how best to suppress or facilitate switching. For the high-stability instruction, the participants were given the task to focus on the figure within the multi-stable stimulus, which was to be primed immediately beforehand, explicitly stating that only the multi-stable stimulus should be rated. The prime was designed to exogenously focus the attention on the given figural region, which should help to hold the multi-stable stimulus steady [32]. The low-stability condition instructed participants to switch the stimulus as often as possible. Since directed attention can influence alternations [19,27], it was specified that if a focused region turned into a figure, attention should be focused back to the ground area to help facilitate another switch. No prime was used in this condition.

The participants were instructed to look at the multi-stable stimulus for at least 5000 ms, after which a notification was presented that the given image should now be rated. With the high-stability instruction, the prime was presented for the first 1300 ms of these 5000 ms, after which it was instantly followed by the stimulus without a mask. After the notification, the stimulus continued to be visible without a time constraint, such that the 5000 ms acted only as a minimum viewing duration. As before, the participants rated liking, valence, and arousal using three separate scales underneath the stimulus. A slider coding for 1–101 was used for liking, while arousal and valence were each assessed using a SAM scale [64] with five subdivisions. Each block was concluded with a rating of perceived exertion and how successful they had been in following the high- and low-stability instructions.

### 3.2. Results

**Figure 4:**
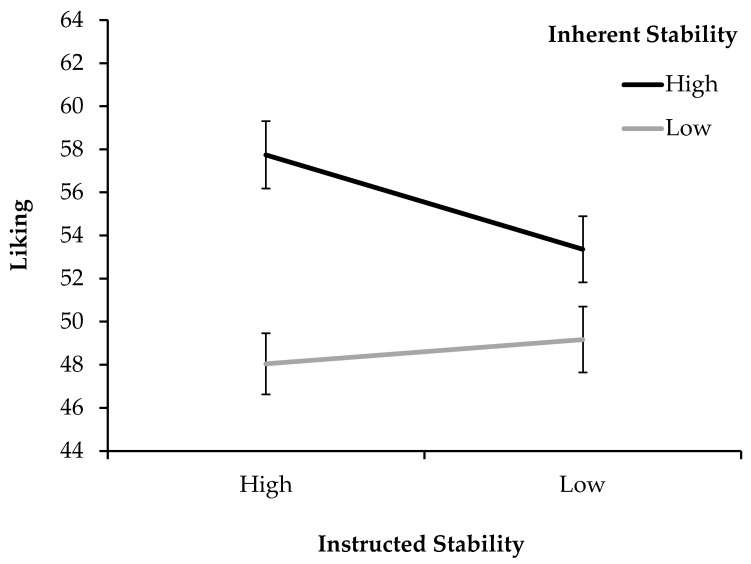
Liking ratings for the high- and low-stability instructions, split by high and low inherent stimulus stability (Exp. 2). Error bars show standard errors of the mean.

As illustrated in Figure 4, the inherently stable figures were liked better than the unstable ones, particularly when the stability instruction was given. In the following, a rmANOVA was conducted with the factors of inherent stability (2) x instructed stability (2) x specific stimulus (11) for the dependent variables of liking, valence, and arousal, including the covariates of gender and participation in the first experiment. All the participants were included in the analysis. Even though Shapiro–Wilk tests showed that arousal and valence were not normally distributed, with respect to Vasey and Thayer [66] stating that the rmANOVA is relatively robust regarding violations of normal distributions, the analysis was left unmodified. In addition, a linear regression model was conducted to predict the covariance of liking and valence.

#### 3.2.1. Liking

When descriptively comparing the averages between conditions, the most notable results were differences regarding stimuli of inherent stability, with overall liking being notably higher for stimuli that are stable (*M_High_* = 55.56, *SD_High_* = 13.42) rather than unstable (*M_Low_* = 48.61, *SD_Low_* = 12.78). The same result, albeit less pronounced, can be seen for instruction, where higher liking was produced by the high-stability instruction (*M_High_* = 52.90, *SD_High_* = 12.77) as compared to the low-stability instruction (*M_Low_* = 51.27, *SD_Low_* = 13.51).

The rmANOVA confirmed both of these effects on liking as significant. For inherent stability, there were large significant effect sizes (*F*(1, 83) = 79.30, *p* < 0.001, *η_p_*^2^ = 0.489), with pairwise comparisons showing that stable stimuli produced significantly higher liking (*p* < 0.001, *M_Diff_* = 6.95, 95% CI [5.39, 8.50]) than unstable ones. Instructed stability showed a significant effect of lesser magnitude (*F*(1, 83) = 4.03, *p* = 0.048, *η_p_*^2^ = 0.046), for which the pairwise comparisons showed that higher likings were produced by the high-stability instruction (*p* = 0.048, *M_Diff_* = 1.63, 95% CI [0.01, 3.24]) compared to the low-stability instruction.

The interaction of inherent and instructed stability was significant and of strong effect sizes (*F*(1, 83) = 26.54, *p* < 0.001, *η_p_^2^* = 0.242). Pairwise comparisons showed that the interaction was carried by the particularly high liking scores for stimuli that were highly stable inherently and also instructed to reverse as little as possible. Within the group with low inherent stability, the stability instruction had no significant effect (*p* = 0.236, *M_Diff_* = 1.13, 95% CI [−0.75, 3.02]). In contrast, within the group with high inherent stability the instruction made a difference (*p* < 0.001, *M_Diff_* = −4.39, 95% CI [−6.36, −2.41]).

#### 3.2.2. Arousal

**Figure 5:**
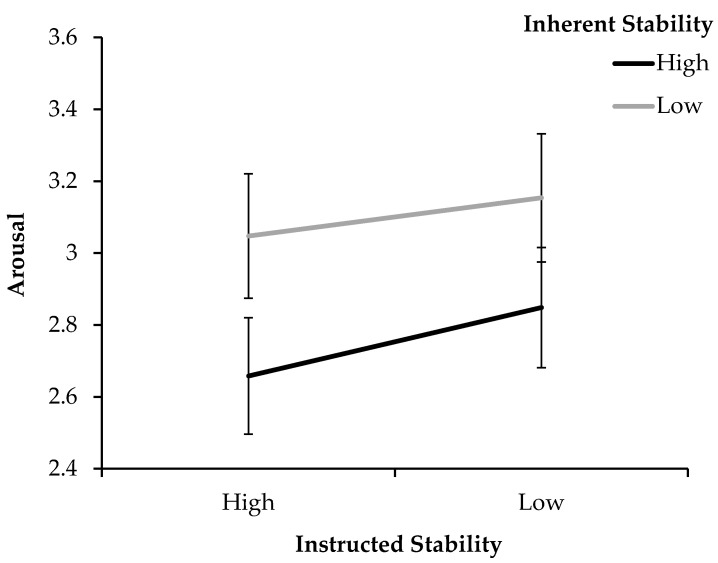
Arousal ratings for the high- and low-stability instructions, split by high and low inherent stimulus stability (Exp. 2). Error bars show standard errors of the mean.

As illustrated in Figure 5, when averaging across conditions, overall arousal was lower for stimuli that were inherently stable (*M_High_* = 2.75, *SD_High_* = 1.46) rather than unstable (*M_Low_* = 3.10, *SD_Low_* = 1.54). Arousal also seemed slightly lower for the high-stability instruction (*M_High_* = 2.85, *SD_High_* = 1.50) than for the low-stability instruction (*M_Low_* = 3.00, *SD_Low_* = 1.55).

The rmANOVA only confirms significance for the effect of inherent stability, which shows a significant effect of large size for arousal (*F*(1, 83) = 39.67, *p* < 0.001, *η_p_*^2^ = 0.323). Pairwise comparisons showed that lower arousal was elicited by stimuli that were more inherently stable (*p* < 0.001, *M_Diff_* = −0.35, 95% CI [−0.46, −0.24]) than unstable ones. Neither an effect of instruction, nor an interaction between the conditions became significant.

To further inspect this interaction more closely, pairwise comparisons for arousal were conducted within each inherent stability group separately, to compare whether the instructions affected them differently. Within the group with low inherent stability, the stability instruction had no significant effect (*p* = 0.236, *M_Diff_* = 0.10, 95% CI [−0.10, 0.31]). In contrast, within the group with high inherent stability, the instruction indeed made a significant difference in arousal (*p* = 0.020, *M_Diff_* = 0.19, 95% CI [0.03, 0.35]).

#### 3.2.3. Valence

**Figure 6:**
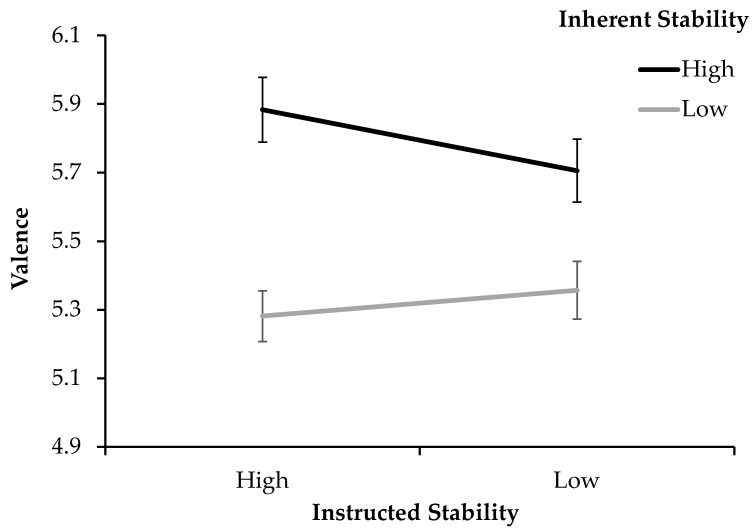
Valence ratings for the high- and low-stability instructions, split by high and low inherent stimulus stability (Exp. 2). Error bars show standard errors of the mean.

Descriptively, when averaging across conditions, the biggest differences in overall valence were produced by inherent stability (Figure 6), with valence being more positive for stimuli that were stable (*M_High_* = 5.79, *SD_High_* = 0.80) rather than unstable (*M_low_* = 5.32, *SD_low_* = 0.66). For instructed stability, valence seems to show no difference between high- (*M_High_* = 5.58, *SD_High_* = 0.70) and low-stability instructions (*M_Low_* = 5.53, *SD_Low_* = 0.72).

The rmANOVA confirms significance for the effect of inherent stability on valence. Inherent stability showed a significant effect of very large size (*F*(1, 83) = 56.66, *p* < 0.001, *η_p_*^2^ = 0.406), with pairwise comparisons showing that significantly more positive valence was produced by stimuli with high inherent stability (*p* ≤ 0.001, *M_Diff_* = 0.47, 95% CI [0.35, 0.60]) rather than low inherent stability. There was no significant effect for instruction on valence. The interaction between inherent and instructed stability reached significance, showing a moderate effect size (*F*(1, 83) = 9.24, *p* = 0.003, *η_p_*^2^ = 0.100).

#### 3.2.4. Liking and Valence Covariation

**Figure 7:**
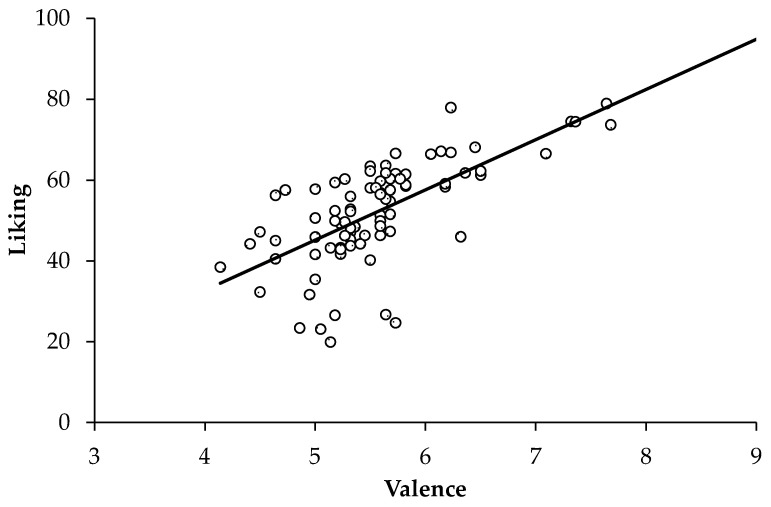
Linear regression of liking as predicted by valence aggregated across all conditions.

To test for the covariation of aesthetics and valence, a linear regression model was conducted to predict overall liking with overall valence, using the aggregates of all stimuli in both conditions (Figure 7). As expected, this model was significant (*F*(1, 82) = 64.34, *p* ≤ 0.001). Valence explained about 43% of the variance in liking (*R*²*_adj_* = 0.433), showing an increase in liking by 12.41 (95% CI [9.33, 15.49] for each rating increase in valence by 1.

#### 3.2.5. Control Variables

When including gender or participation in the first experiment as a between-subject factor for the rmANOVA above, neither showed a significant effect. In the rmANOVA, the stimulus factor reached significance for liking (*F*(7.66, 636.18) = 34.91, *p* ≤ 0.001, *η_p_*² = 0.296). The variance between individual multi-stable stimuli in liking, for Exp. 2 as well as Exp. 1, is illustrated in Appendix A. To control for perceived exertion and subjective adequacy in task completion, multiple linear regression models were conducted, which were used to predict liking, arousal, and valence in each block. None of these models became significant.

**Figure 8:**
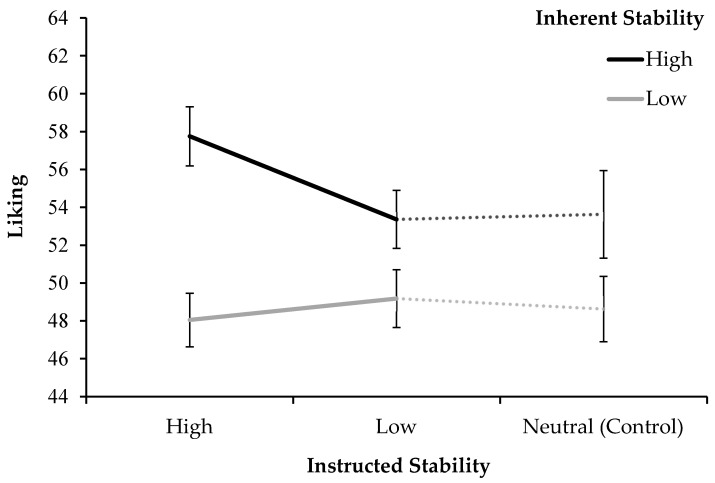
The mean liking scores of multi-stable stimuli from both experiments, comparing high and low instructed stability (Exp. 2) with the control of no instruction (Exp. 1), split by inherent stimulus stability. Errors bars represent standard errors of the mean.

The liking ratings across both experiments are graphed in Figure 8. The figure compares the instructions of high and low stability (Exp. 2) with no instruction (Exp. 1) as a control condition, each data point being split by high and low inherent stimulus stability.

### 3.3. Discussion

The second experiment again shows that for multi-stable stimuli, higher stability elicits higher aesthetic appreciation. This was the case when stability was achieved through inherent stimulus properties, as well as for active perceptual control, albeit to a lesser extent. The main driver of aesthetic appreciation seems to be inherent stimulus stability, which produced by far the largest effects of higher liking, more positive valence, and lower arousal. The instruction to make a stimulus more stable by controlling perceptual switching boosted the aesthetic appreciation of stimuli that were inherently stable to begin with, but did little for inherently unstable stimuli. Figure 8 illustrates the specificity of this interaction in the case of liking, which was likewise demonstrated for arousal.

Again, a processing fluency hypothesis could explain the general tendency whereby higher stability, through higher processing ease, would produce a bias towards positive stimulus assessment (hence higher liking and positive valence). The decline of arousal with higher stability can likewise be explained with the notion that higher fluency requires fewer resources and goes together with relaxation afforded by easier processing. Because of this, we predicted main effects both for inherent properties and instruction. Any stability that resulted from the manipulations, irrespective of its source, should positively influence aesthetic appreciation, and vice versa for instability.

Importantly, we also assumed that for a stimulus which already has the tendency to switch easily, an instruction to enhance these switches (as opposed to preventing them) might be easier to implement and hence more effective. Thus, we predicted an interaction if the conditions point in the same direction, in which case any resulting in (in)stability would be further amplified. However, this interaction was only shown for the case of combining two high-stability conditions, not for instability. Whereas fluency can account for the large positive effect for high stability, it would also predict a negative outcome for the inverse case. Instead, for the combination of two sources of instability, unexpectedly, the instruction had no additional negative effect on appreciation. Thus, fluency cannot fully account for these findings.

## 4. General Discussion

The object of the present study was to investigate the appeal of multi-stable figures. On the one hand, we are fascinated by figures that assume one perceptual organization some of the time, and then inexplicably switch to an alternate perceptual organization, but never make both available at the same time. Could this switching be related to their aesthetic qualities? On the other hand, one of the dominant theories of aesthetic appreciation, processing fluency, explains aesthetic liking by the ease with which a stimulus can be processed. According to the notion of fluency, perceptual instability should reduce liking. We explored the role of stability in the intuitive appreciation of multi-stable stimuli by presenting multi-stable figures that were either comparatively stable or highly unstable. We also instructed observers to willingly hold on to one perceptual organization or to make the organization switch as much as they could. The results are mostly straight-forward: stability, be it stimulus-inherent or be it instilled by will power, produced higher liking scores. In contrast, instability was generally associated with reduced liking. We discuss several interpretations of these findings, as well as a number of caveats.

### 4.1. Does Stability Produce Fluency?

When analyzed through the lens of processing fluency, every perceptual reorganization or switch can be understood as a disruption of sensory processing, just as the intrinsic ambiguity that lies in multi-stability can be thought of as an obstacle which stands in the way of an easy, clear, and fluent interpretation of a stimulus. Note that even if the observer does not notice a switch, the underlying perceptual organization process is likely less fluent. We argue that with increasing instability, the pressure for the visual system to maintain a given organization is increased. With it, disruption is more likely, and feelings of ambiguity get more pronounced. Through this mechanism, in both experiments, higher stimulus stability may have produced a combination of higher liking, more positive valence, and lower arousal. Generally, this effect seems to be perceptual initially but can in some circumstances be modified by active top-down control. Since fluency is generally associated with relaxed and positive emotions in the viewer, we interpret the combination of liking, relaxation, and positive valence as bearing witness to fluency as well as aesthetic appreciation. Thus, across our two experiments, it seems safe to say that the perceptual instability of simple multi-stable stimuli, fascinating as it may be, is detrimental to their aesthetic quality.

Whereas the overall trend within the data can be explained through processing fluency, some smaller parts of the results pose a challenge to the theory. For example, if switches truly are detrimental to fluency, removing perceptual switches altogether should produce the highest liking scores. However, in the first experiment, while isolated figures were descriptively liked more than multi-stable stimuli, the trend failed to reach significance. Note, however, that one might argue that the liking scores of one of the two objects contained in each multi-stable stimulus may not be directly comparable to the compound stimulus. To be sure, one would have to present stimuli that contain both figures side by side such that they cannot reverse.

### 4.2. Extending Processing Fluency by Expectation and Salience

In their landmark paper on processing fluency, Reber et al. [8] elaborated on why the theory might not necessarily always predict that the simplest stimulus is also the most beautiful. The fact that observers often prefer complex stimuli over simple ones could be integrated into fluency if one accounts for two influences: the effect of fluency attribution, as well as the expected processing fluency.

Firstly, once fluency leads to pleasant viewer states, this pleasantness still has to be attributed to the stimulus. This attribution is not guaranteed to happen and can indeed be disrupted. Accordingly, Reber et al. [8] posit that if a stimulus is overtly simple, the influence of fluency becomes more salient, such that an observer is more likely to recognize that their positive state indeed results from easy processing itself, not from the stimulus. In this case, the attribution of appreciation to this given stimulus is less likely to happen. Secondly, they posit that expectation of fluency influences the intensity of the effect: when a stimulus initially seems like a challenge, but then is processed fluently despite this expectation, it will elicit more appreciation. Conversely, when observers are presented with a simple stimulus, which they expect to be able to process easily, this should diminish the positive effect of fluency on liking.

Structurally, every isolated figure makes it obvious to the viewer that it will be easy to process. Thus, even though their fluency is objectively much higher, this advantage may not fully come to the fore because this fluency is already expected and is made salient to the observer. Now, some multi-stable stimuli are relatively stable (and hence easy to process), while others are relatively unstable (difficult to process). And since the observer initially cannot tell their stability, they should categorically expect a bigger challenge for any multi-stable stimulus. If the stimulus then turns out to be relatively stable, contrary to prior expectation, the resulting fluency would be amplified. Additionally, since the salience of this fluency would also be lower, the liking would indeed be attributed to the stimulus. Thus, expectation and salience would obstruct fluency effects somewhat for isolated figures, but enhance effects for multi-stable stimuli. This could explain why, despite a significant difference in objective fluency, isolated figures produced only slightly higher liking scores, if at all, than the multi-stable stimuli of high stability.

Another challenge for the fluency explanation is a result from the second experiment, where the stability instruction only showed an effect in the condition where it enhanced an already stable stimulus. This poses two questions: why did the instruction only produce large effects in an interaction, and why did this interaction only work for stability, but not for instability? We predicted a symmetric interaction such that the instruction to prevent/enhance switches would always increase/decrease liking, respectively. The finding that instructions only showed comparatively small main effects, but large interactions, might be explained by the simple fact that the instruction was only potent enough to produce noticeable stability if it was added to a comparatively stable stimulus. The instruction to facilitate switches may simply not have worked, or preventing switches in an unstable stimulus may not have been feasible for the participants. However, this would still not answer the question of why the interaction produced large effects for a combination of high stability, but not for instability.

A caveat is that the high-stability instruction was also assisted by the help of a prime, for which there was no equivalent in the low-stability instruction. Such an equivalent can of course not exist. It is conceivable that it was not the instruction, but merely the prime per se that induced fluency. However, one would still need to explain why any potential benefits only increased appreciation for stable, but not for unstable, stimuli. It might just be that for unstable stimuli any potential fluency of a prime was quickly compensated by subsequent perceptual switches. Be this as it may, inherent stimulus properties seem to be the biggest driver behind any aesthetic effects, while external manipulations of the observer’s intention play a more modest role.

### 4.3. The Dual Face of Ambiguity

The processing fluency framework has shown its limitations. Until now, we mostly conceptualized ambiguity within multi-stability as an obstacle towards aesthetic appreciation. However, while some studies showed that removing ambiguity from a multi-stable stimulus increases its aesthetic appreciation [67,68], others found it to be decreased by ambiguity removal. Nicki et al. [69], for example, showed that for the duck/rabbit figure, when presenting either the unambiguous version of the duck or the rabbit in isolation, the single figures produced lower liking and interest than did the original multi-stable image. Parallel to this, Noguchi [70] showed that aesthetic preference rises in direct relation with a figure’s perceived illusion intensity, such as when investigating Oppel–Kundt grids or Helmholtz radials. This demonstrates that in some cases, aesthetic appreciation is increased when a stimulus gets clearer, simpler, and better defined, whereas in other cases, stimuli are liked more as they get more complex and ambiguous. Apparently, some stimuli are actively preferred when they obscure a clear interpretation, thereby revealing to the observers that their own sensory process is not as trustworthy as they often believe it to be.

In this context, ambiguity has to be understood as having a two-fold effect. It can lower fluency, and it can also increase the potential for insight and thereby enhance liking. For instance, Belke et al. [45] distinguished between fluency and mastery in portraits. During an in-depth exploration of the stimuli by the observers, their appreciation for fluent portraits remained flat over time, whereas portraits with higher mastery increased in liking over time. The authors claim that this was the case because only the latter group could properly elicit and benefit from cognitive stimulation. Graf and Landwehr [71] tried to reconcile these two paths towards aesthetic appreciation in their pleasure–interest model. They posited that when in a default state, an automatic, passive, fluency-driven path guides stimulus evaluation. However, when an unexpected fluency discrepancy is triggered, a second process may take over, which in contrast is actively controlled and cognitively driven, with the aim of reducing any disfluency. They hypothesized that when confronted with a discrepancy in fluency during processing, the observer’s capability and motivation subsequently mediates whether the necessary resources are actually recruited, in order to shift from a passive into a more demanding active processing pathway. For this shift, an observer needs to have the incentive for cognitive enrichment.

However, even in the case where a multi-stable image in one of the experiments might trigger a fluency discrepancy, the inherent potential for cognitive enrichment within our stimuli should be decidedly low. They certainly do not betray the mastery of a great painter or visual artist. Due to the nature of their multi-stability, they likely induce an Aha effect when the observer discovers the alternate interpretation of the stimulus. However, to be able to attribute perceptual stability to fluency, we designed the stimuli to be both stylistically simple as well as homogenous in nature and therefore had equal, comparatively low ceilings for potential mastery. Moreover, since the type of multi-stability functionally was the same across every stimulus in the study, the Aha effect is likely to be diminished with repeated presentation of structurally similar images. After one or two perceptual switches, the entire potential for cognitive enrichment could be exhausted.

In contrast, a more complex multi-stable painting like “Forever Always” by Octavio Ocampo, when compared to Rubin’s vase, holds more potential for insight. Here, the insight exceeds the discovery of a second interpretation, because the observer can subsequently also trace how exactly a given section can be assembled into two different Gestalts. Thus, multiple smaller insights also lie in the complex discovery of how an arm of the guitarist can also convey the nose of an old man or how a turban can be seen as an eyebrow, whereas our stimuli simply present the viewer with plain white or black areas. Increasing the instability in a more complex multi-stable stimulus might therefore involve different processes not accessed by our stimuli. Caution should be used when extending the results to very complex multi-stable images or to ambiguous stimuli in general.

Even when keeping these dual-process implications in mind, our findings nonetheless have implications for practical use cases. Most notably in the realm of design, when for example devising a company logo or a web interface with a multi-stable element, it is safe to say that this visual ambiguity comes with a risk. It should not be taken for granted that reversibility of a figure is inherently pleasurable; instead, this property may rather be understood as a means to an end for cognitive enrichment. If an ambiguous element like multi-stability obscures fluent processing, it should involve a worthwhile cognitive payoff. Otherwise, a choice that intends to make a design more engaging might simply make it less easily digestible and thereby less appealing. Similarly, the result that visual instability can be detrimental to aesthetic appreciation might also inform research in the realm of technical image processing, such as when deciding to what extent the loss of visual clarity and stability during video compression schemes might be tolerable [72].

## 5. Limitations

Given the simplicity of our stimulus material, which enabled us to exercise precise stimulus control, we may have conducted our research in the realm of very low aesthetic value. Compared to a Rembrandt or a Monet, most observers might have given our stimuli very low liking scores. It is thus fair to ask if our results will generalize to more masterly works of art. The problem here is that in the classic western canon of paintings, only a few multi-stable works come to mind, such as Salvador Dali’s “Slave Market with the Disappearing Bust of Voltaire”, or some of M. C. Escher’s works. Even the latter, however, cannot be easily varied in their intrinsic stability and thus could not have served as stimuli for our experiments. In the context of stimuli with more potential for cognitive enrichment, differentiating aesthetic pleasure from interest could become relevant again. For the present study, mild pleasantness and relaxation can reasonably be viewed as aesthetic appreciation for a simple fluency-type stimulus, but this conceptualization cannot necessarily be generalized onto other forms of appreciation, which more sophisticated works of art are able to elicit.

As already noted, some limitations of the present study lie in the stimulus material itself. Since both studies used the same set of images, we cannot guarantee that any effects we showed are generalizable to more sophisticated artworks. Also, even though the figure–ground functionality of the stimuli was validated by Kuo et al. [59], since many figures also had outer contours that were not fully integrated into a connecting border of the other part of the figure, the switching mechanism between the two areas might have been compromised in some instances. This is especially relevant, as we determined the stability of a stimulus solely through a subjective assessment of the observers. In contrast, an experimental setup where participants have to press a button every time the stimulus interpretation switches could have provided a more robust measure for the relative dominance of a given Gestalt. This setup would make it easier to track if the stability manipulations indeed work as intended. Additionally, it would make the usage of a prime in the high-stability instruction dispensable, which in the current design might have involved the risk of confounding effects. However, while a more direct stability measure would have led to more data, it would have also been more taxing for observers. Thus, we did not record the numbers of subjective switching experiences, as we feared that this would either compromise the aesthetic assessment if measured simultaneously to liking scores or it would disproportionately increase the runtime of the experiment if placed in a separate block.

## 6. Conclusions

We have shown that multi-stable images are not appreciated aesthetically for their characteristic ability to switch from one perceptual organization to another. In fact, both their relative intrinsic stability and their instructed stability increased aesthetic liking. Instability was detrimental to aesthetic appreciation. Higher processing fluency of multi-stable images that remain comparatively stable is a likely explanation for this effect.

## Figures and Tables

**Figure A1:**
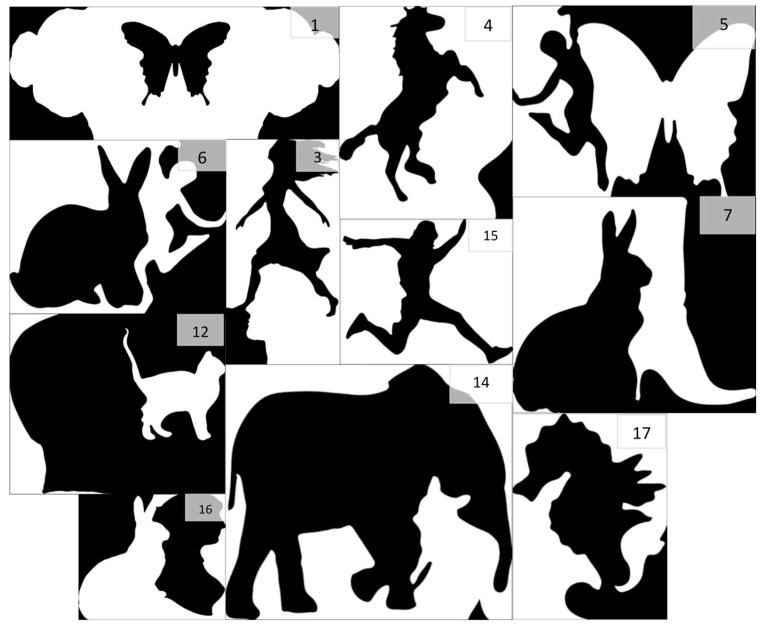
The 11 multi-stable stimuli of the low-stability group that were rated in Table A1.

**Figure A2:**
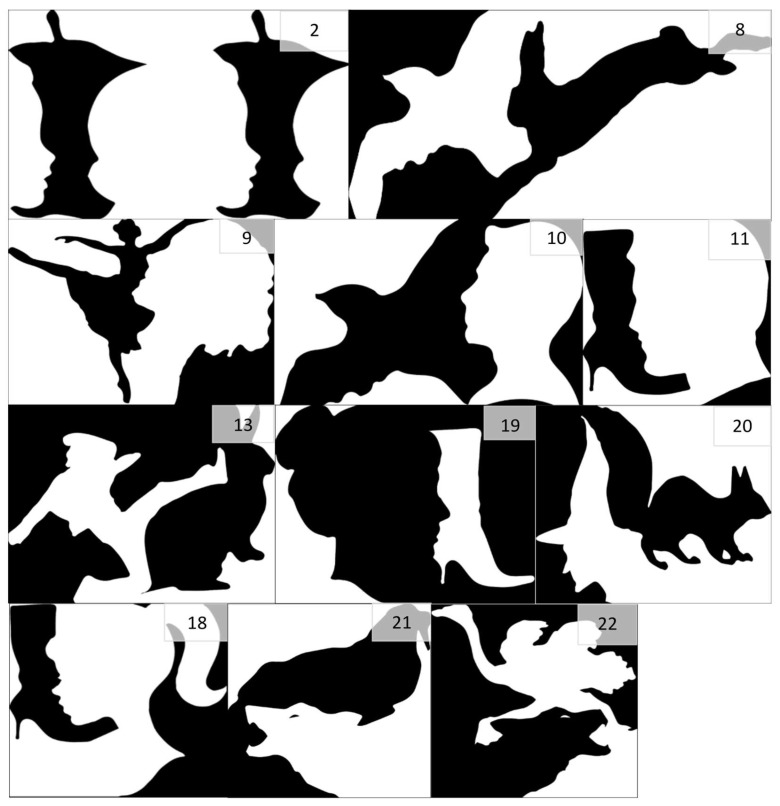
The 11 multi-stable stimuli of the high-stability group that were rated in Table A1.

**Figure A3:**
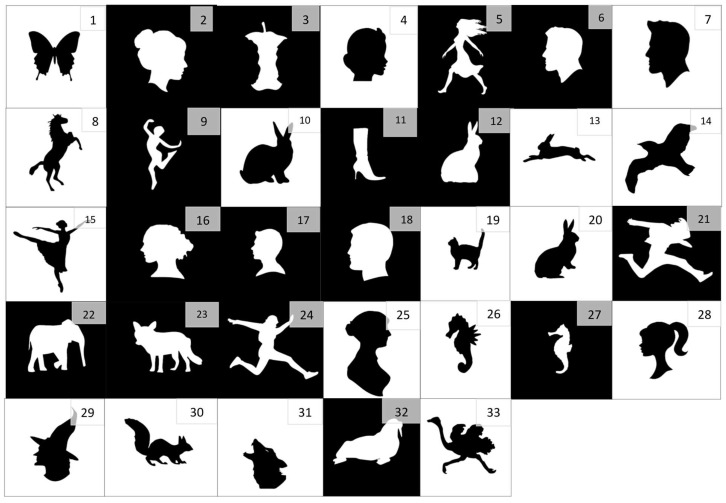
All 33 isolated stimuli. Their associated liking scores are listed in Table A3.

**Figure A4:**
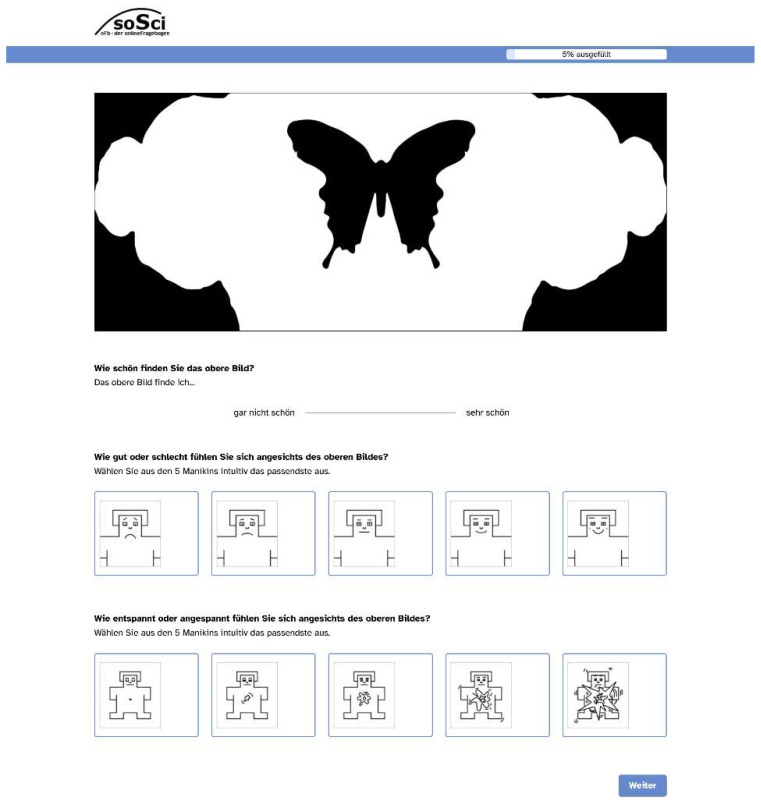
A slide of the questionnaire is illustrated, including the original questions in German. The question for liking reads “How beautiful do you judge the image above to be? I find the image above to be…”, with the slider being labeled with “not beautiful at all” to “very beautiful”. The SAM scale for arousal reads “How relaxed or aroused do you feel regarding the image above? Intuitively pick the most fitting out of the 5 manikins”. The SAM scale for valence reads “How good or bad do you feel regarding the image above? Intuitively pick the most fitting out of the 5 manikins”. On a separate slide from the aesthetic measures, the original stability question in German read “Wie häufig kippen die Figuren im oberen Bild von Vorder-zu Hintergrund und andersherum? Verglichen mit den anderen Bildern kippen die Figuren… (nie—sehr häufig)”. Translated into English, the question reads “Relative to the other images—how often do the figures in the image above switch from foreground to background and vice versa?”.

## Data Availability

Original data are available in an Open Science repository: https://osf.io/msr67/ (accessed on 3 April 2025).

## Supplementary Materials

Supplementary material is available in an Open Science repository: https://osf.io/msr67/ (accessed on 3 April 2025).

## Appendix A

### Appendix A.1. Stimuli with High Inherent Stability

**Table A1:** Liking scores for individual stimuli with high inherent stability. All corresponding stimuli are illustrated below. Stability rankings were only assessed in Exp. 1. Note that stability codes for frequency of switches; therefore, a higher score shows higher instability. Liking scores for Exp. 2 represent the mean values across both stability instructions.

		Exp. 1		Exp. 2	
Stimulus	Stability	M	Std	M	Std
1	57.5	64.8	26.1	68.9	17.6
3	61.8	63.1	24.3	62.9	18.3
4	63.8	50.1	24.0	54.7	19.4
5	68.9	61.6	25.4	63.1	19.2
6	64.3	54.7	22.8	51.5	19.9
7	64.8	44.1	22.6	50.2	17.6
12	66.5	40.9	29.9	46.3	21.8
14	65.2	60.9	25.5	60.7	19.1
15	43.9	42.6	23.9	43.2	16.8
16	68.9	45.5	23.1	48.4	16.5
17	64.1	61.4	23.6	61.2	18.7

### Appendix A.2. Stimuli with Low Inherent Stability

**Table A2:** Liking scores for individual stimuli with low inherent stability. All corresponding stimuli are illustrated below. Stability rankings were only assessed in Exp. 1. Note that stability codes for frequency of switches. Thus, a higher score corresponds to higher instability. Liking scores for Exp. 2 represent the mean values across both stability instructions.

		Exp. 1		Exp. 2	
Stimulus	Stability	M	Std	M	Std
2	71.8	42.9	27.2	44.7	20.6
8	73.7	53.6	26.3	53.9	18.7
9	70.3	71.3	22.4	70.7	19.1
10	72.8	54.3	22.0	50.9	16.6
11	69.6	39.6	22.5	38.7	16.1
13	70.2	40.5	23.2	37.0	19.1
18	69.3	49.1	26.2	54.9	18.2
19	74.1	43.8	24.6	46.0	17.8
20	69.8	45.8	22.2	40.4	20.3
21	70.7	48.4	24.1	48.8	22.7
22	69.5	45.7	28.4	48.6	22.0

### Appendix A.3. Isolated Figures

**Table A3:** Liking scores for isolated figures. All corresponding stimuli are illustrated below.

Stimulus	M	Std	Stimulus	M	Std
1	71.8	21.7	17	43.6	22.0
2	64.5	21.6	18	44.1	18.9
3	40.9	26.6	19	71.5	21.4
4	60.8	21.6	20	63.8	19.7
5	58.6	22.3	21	43.3	24.7
6	49.9	20.6	22	68.2	21.4
7	46.0	21.9	23	64.2	21.4
8	60.2	21.5	24	47.8	21.5
9	56.1	23.0	25	46.5	20.0
10	61.9	20.2	26	67.8	20.8
11	43.2	24.4	27	66.9	20.7
12	54.5	23.8	28	62.5	27.2
13	57.6	21.5	29	33.4	21.7
14	59.7	20.9	30	67.1	20.2
15	71.9	21.0	31	56.3	25.9
16	55.8	21.2	32	56.1	22.9
			33	49.3	22.9
